# Stem Cell Theory of Cancer: Implications for Translational Research from Bedside to Bench

**DOI:** 10.3390/cancers14143345

**Published:** 2022-07-09

**Authors:** Shi-Ming Tu, Sunny R. Singh, Konstantinos Arnaoutakis, Sindhu Malapati, Sajjad A. Bhatti, Aron Y. Joon, Omar T. Atiq, Louis L. Pisters

**Affiliations:** 1 Division of Hematology/Oncology, University of Arkansas for Medical Sciences, Little Rock, AR 72205, USA; srsingh@uams.edu (S.R.S.); karnaoutakis@uams.edu (K.A.); smalapati@uams.edu (S.M.); sabhatti@uams.edu (S.A.B.); otatiq@uams.edu (O.T.A.); 2 Department of Bioinformatics, The University of Texas MD Anderson Cancer Center, Houston, TX 77030, USA; ayjoon@mdanderson.org; 3 Department of Urology, The University of Texas MD Anderson Cancer Center, Houston, TX 77030, USA; lpisters@mdanderson.org

**Keywords:** translational research, heuristic research, scientific method, drug development, therapy development, data reproducibility, genomic medicine, integrated medicine

## Abstract

**Simple Summary:**

To find the cause of our current failures in drug development and data reproducibility, we may need to search no further than the basic premises of translational research. To enhance cancer research and cancer care, we need to be diligent in the proper application of the scientific method. We postulate that a stem cell theory of cancer embraces genomic medicine and empowers integrated medicine. It alludes to a unified theory of cancer that may advance cancer research by emending translational research and enforcing the scientific method. It may enhance patient care by enabling targeted therapy and employing multimodal therapy, so that we treat the whole cancer and heal the whole patient.

**Abstract:**

A stem cell theory of cancer considers genetic makeup in the proper cellular context. It is a unified theory of cancer that unites the genome with the epigenome, links the intracellular with the extracellular, and connects the cellular constituents and compartments with the microenvironment. Although it allies with genomic medicine, it is better aligned with integrated medicine. In this perspective, we focus on translational research in cancer care. We expose some intrinsic fallacies in translational research when it relates to the basic principles of the scientific method in the care of patients with genomic medicine versus integrated medicine. We postulate that genomic medicine may be at the root of many failed efforts in drug development and data reproducibility. We propose an alternate heuristic approach that may expedite the development of safe and effective treatments and minimize the generation of unproductive pharmaceutical products and nonreproducible experimental results. Importantly, a heuristic approach emphasizes the role of a pertinent scientific theory and distinguishes therapy development from drug development, such that we discover not only useful drugs but also better ways to use them in order to optimize patient care and maximize clinical outcomes.

## 1. Introduction

“*Whether or not you can observe a thing depends upon the theory you use. It is the theory which decides what can be observed*”.

Albert Einstein

Nowadays, most researchers and clinicians have accepted the ethos of translational research with great enthusiasm. We tend to translate basic research from the laboratory (bench) to patient care in the clinic (bedside) rather than the other way around. We believe that translational research performed in this manner is conceptually solid and practically sound; it assures efficiency and efficacy in our provision of superior health care.

However, when we are encumbered by innumerable ineffective and unsafe treatments from drug development [1,2], and mystified by abundant nonreproducible data and unverifiable results from influential experiments [3,4,5], perhaps we should reexamine the merits and reevaluate the promises of translational research. When it becomes incumbent upon us to convert incremental clinical advancements to exponential breakthroughs and marginal clinical improvements to meaningful patient benefits, perhaps it is time to return to the fundamentals of scientific research and revisit the principles of the scientific method to reappraise translational research.

In this perspective, we focus on the implications of a stem cell theory of cancer for translational research in cancer care. We expose some intrinsic fallacies in translational research when it relates to the scientific method for the care of patients with genomic medicine versus integrated medicine. We postulate that genomic medicine may be at the root of many failed efforts in drug development and data reproducibility. We propose an alternate heuristic approach that may expedite the development of safe and effective treatments and minimize the generation of unproductive pharmaceutical products and nonreproducible experimental results. Importantly, a heuristic approach, as predicated by the scientific method, emphasizes the role of a pertinent scientific theory and distinguishes therapy development from drug development, such that we discover not only useful drugs but also better ways to use them in order to maximize patient care and optimize clinical outcomes.

## 2. Scientific Method

Even though we preach the scientific method, it is evident that many of us do not practice it. Perhaps when we do adopt and adhere to the scientific method, we could minimize the failures in drug development and mitigate a dilemma in experimental reproducibility. 

According to the scientific method, the first step in our scientific endeavor is to make a reliable and relevant clinical observation [6,7,8]. If a treatment shows extraordinary benefit in the clinic and the resulting hypothesis is pertinent, testing the hypothesis and understanding its mechanism of action is beneficial because the resultant knowledge will help us select the right patients and apply it in the right settings. However, if a treatment shows no clinical benefit at all, then we need to question the prevailing hypothesis or formulate a better one. Otherwise, testing the wrong hypothesis is moot and learning about its putative mechanism of action can be self-fulfilling and self-serving.

Therefore, it is crucial that we make insightful clinical observations and pose judicious scientific hypotheses to start a promising and rewarding scientific journey. It behooves us to acknowledge that successful clinical observations are pre-requisite for successful biological research, and that preclinical research is essential for the generation of successful clinical treatments. Indeed, translational research should be a bidirectional process and would be most rewarding when there is a continuous exchange between bedside and bench.

With the correct scientific method, we prevent research that is likely to be misguided and misleading and avoid treatments that may be ineffective or intolerable. With a proper itinerary, we avoid scientific misdirection and misadventures.

## 3. Translational Research

Translational research is potentially problematic in biomedicine because, in many respects, biomedicine is an inexact science. It is challenging to “translate” something that is inherently intricate, erratic, and nuanced. 

Another dilemma of translational research is that in a dynamic system and mutable network, as opposed to a static event or isolated target, timing and context matter. Otherwise, the message and meaning will be lost in translation.

In our standard reductionist mode of scientific research and conventional deductive view of a logical world, is it proper and appropriate to translate word by word or word for word? Unless we see the whole elephant, are we at risk of not knowing what it is really like when we translate only the parts that we (choose or happen to) touch but cannot see?

One way to make translational research relevant is to make sure that it adopts and adheres to the scientific method [6,7,8]. However, there could be a hidden obstacle to this objective in the very nature of translational research: The scientific method requires that we make a seminal observation and formulate a pertinent hypothesis about the observation first. Only then do we design experiments to test the hypothesis and interpret the results.

Therefore, moving from bench to bedside as the first step in the process of translational research seems to be the reverse of what the scientific method requires. In other words, the observations we make in the laboratory should be the results of testing hypotheses, not for the purposes of generating them. When we investigate whether adding factor A or removing target B eliminates or cures cancer X in a cancer cell line or animal model, we determine whether factor A or target B plays a role in the pathogenesis of cancer X in the laboratory. However, when we assume that manipulation of these factors is operative in the real world, we render the results of our experiments hypothesis generating rather than hypothesis testing. 

Ultimately, a hypothesis (in biology, if not in physics) may be flawed or false without a reliable or pertinent observation. Often enough, an occurrence translated from the laboratory, such as the manipulation of factor A or target B clinically benefitting patients with cancer X, has not been observed in the clinic. Importantly, this version of translational research is likely to be erroneous, because it runs counter to the basic premises of the scientific method.

## 4. Heuristic Approach

A heuristic approach is a practical method of problem solving or self-discovery based on evidence and experience that is sufficient and efficient for the purposes of reaching a specific objective. We predict that a heuristic approach that embraces the principles of the scientific method may have tangible advantages over the currently predominant reductionist mentality in cancer research. We anticipate that the goals of heuristic research may be more realistic and the results more fruitful compared with those derived from traditional translational research when it concerns drug development and data reproducibility.

It is essential for us to distinguish this heuristic approach from translational research. The distinction is critical because the heuristic approach requires that we apply the scientific method in the proper sequence and in the appropriate manner. 

Contrary to conventional wisdom regarding translational research, in which we pose a hypothesis based on preclinical evidence in the laboratory and then test the hypothesis in the clinic, in heuristic approach we formulate the hypothesis based on prevalent and pertinent (especially extraordinary) clinical observations and then test the hypothesis in the laboratory (to learn the mechanisms of action) and in the clinic (for drug and therapy development). 

Indeed, this is how we discovered androgen-deprivation therapy to treat prostate cancer [9] and chemotherapy to cure testicular cancer [10]. Although clinical and laboratory research is inherently bidirectional, we advocate that the first step in translational research should be bedside to bench in an effort to maximize impact and optimize outcome. 

Otherwise, we create two major problems in cancer care: (1) We produce countless ineffective and unsafe cancer treatments. This is a waste of our resources, e.g., money, time, and effort. (2) We also produce myriad nonreproducible experimental data and unverifiable results. This is an embarrassment to our scientific integrity and an insult to our scientific credibility.

### 4.1. Drug Development 

It is noteworthy that the overall failure rate in drug development is over 96%, including a 90% failure rate during clinical development [2]. Importantly, about 2 out of every 5 drugs that reach “confirmatory” phase 3 trials still fail to earn approval for the indication being investigated. The overall probability of success for drug development is particularly dismal in the field of oncology (3.4%) compared with vaccines (33.4%) [11].

Reasons for failure of clinical trials to eventually result in FDA approval include lack of efficacy, issues with safety, insufficient funding to complete the study, as well as other factors, such as problems with patient recruitment, enrollment, and retention [1].

### 4.2. Data Reproducibility

Recently, Errington et al. demonstrated that only 46% of attempted replications confirmed the original published findings [5]. Their results reaffirmed previous reports in which more than 70% of researchers have tried but failed to reproduce another scientist’s experiments, and more than half have failed to reproduce their own experiments [12]. In addition, reviews of preclinical biological studies suggest that only 20–25% of findings were reproducible [13,14], and of drugs and other treatments targeting cancer only 11% of findings from “landmark” studies were replicable [15]. 

Proposed fixes for a crisis in data reproducibility include “blinding, bigger sample sizes, greater statistical rigor”. Instead of soul-searching and self-reproaching, Baker et al. recommended “more proofs and fewer claims, spirit of data sharing, and culture of self-correction” [12].

## 5. Genomic Medicine

Genomic medicine involves use of genomic information in clinical care (e.g., for diagnostic and therapeutic decision making). Its palpable influence on health outcomes and policy implications is undeniable.

Perhaps our captivation by genomic medicine and enamor with translational research have contributed to a plethora of failures in drug development and data reproducibility. 

Let us examine the sequelae of translational research from the perspective of genomic medicine. According to the genetic theory of cancer and a multistep model of carcinogenesis [6,7], acquisition and accumulation of genetic mutations initiate and promote the development of cancer. We observe specific mutations and assume that they cause the formation of certain malignant tumors. Targeting those specific mutations, such as BCR-ABL in chronic myelocytic leukemia and PML-RAR-alpha in acute promyelocytic leukemia (APL), has certainly provided clinical benefits and validated the basic principles of a genetic origin of cancer.

Except that a vast majority of genetic mutations may be effects rather than causes of cancer. According to Forrest et al. [16], the chance that a genetic variant (euphemism for mutant) will be linked to a disease diagnosis is relatively low—only about 7%. Therefore, it is expected that there will be many passenger mutations and few driver mutations in a malignant tumor. In fact, we have discovered that some of those very drivers are also present in benign tumors and nonmalignant tissues [17,18,19,20,21]. Yet, we have designed treatments and experiments for the purpose of hitting those purported cancer targets and fixing the putative driver mutations. When we invest in this strategy of genomic medicine despite its inherent redundancy and irrelevancy, we risk producing failed pharmaceutical products and faulty experimental results. 

### 5.1. Favorable Mutations 

We are attuned to think that genetic mutations are bad because they make cancer what it is—menacing and deadly. However, certain genetic mutations in certain tumors seem to confer improved clinical outcomes [22,23]. For example, five-year survival of patients with gliomas containing IDH mutation or ATRX mutation (IDH and ATRX are mutually exclusive) and 1p/19q co-deletion is up to 80%, whereas those with gliomas not containing IDH or ATRX mutation and without 1p/19q codeletion is 5% [22]. Similarly, certain mutations such as SF3B1 confer improved prognosis in myelodysplastic syndrome (MDS) [23].

### 5.2. Innocuous Mutations 

Although some studies suggested that an increased number of driver mutations adversely affects prognosis in MDS [24,25], others indicated just the opposite [26]. Perhaps the type of mutations (e.g., TP53, EZH2, RUNX1, ASXL1, ETV6) also matters, i.e., quality trumps quantity [27]. It is imperative for us to separate driver from passenger mutations, seminal events from bystander effects, and vantage point from background noise in the pathogenesis of cancer. Indeed, when some of the supposedly driver mutations (e.g., ASXL1) are also detected in clonal hematopoiesis of indeterminate potential (CHIP) [28], perhaps it is not just the number and type of mutations that count. When it concerns MDS and CHIP, it seems that even though genetic content may be critical, cellular context is paramount. 

### 5.3. Somatic Hypermutations 

Another contradiction in genomic medicine relates to the improved prognosis of mutated compared with unmutated IGHv status in chronic lymphocytic leukemia (CLL) [29]. Again, this may be explained by cellular context [30]. For example, VH mutations tend to occur in differentiated CD38+ T and B cells from which a mutated CLL originates [31]. We propose that cellular origin has prognostic and predictive implications despite the genetic makeup. Consequently, malignancies derived from more differentiated progenitor cells entail improved prognosis that may or may not be dictated by specific mutations, as well as improved outcomes that may or may not be affected by specific treatments. 

Similarly, hypermutations may be associated with better prognosis, rather than worse prognosis, in microsatellite instability-high vs. stable (MSI-H vs. MSS) gastric and colorectal cancers [32,33,34,35,36]. For example, patients with stage II MSI-H colorectal cancer have a relatively good prognosis and do not benefit from adjuvant chemotherapy [37]. We speculate that the cell of origin, i.e., cellular context, accounts for the prognostic and predictive implications of these malignant tumors: MSI-H tumors are more likely to derive from defective progeny differentiated cells, in which DNA repair defects are necessary to incur malignant transformation, than from progenitor stem cells, in which asymmetric division and aneuploidy are more likely to occur and hypermutation becomes less consequential [6,7,38].

## 6. Integrated Medicine

A stem cell theory of cancer postulates that there is a hierarchical order of progenitor cancer stem cells and progeny-differentiated cancer cells. It predicates that cancer possesses intrinsic stem-like properties and pathways. In many respects, cancer is a stem-cell disease. A malignant cell mirrors, if not mimics, a normal stem cell. Different cancer subtypes have distinct cellular stem-ness origins. 

A stem cell theory of cancer considers genetic makeup in the proper cellular context. It is a unified theory of cancer that unites the genome with the epigenome, links the intracellular with the extracellular, and connects the cellular constituents and compartments with the microenvironment [6,7,39,40,41,42]. Although it allies with genomic medicine, it is better aligned with integrated medicine.

We define integrated medicine as a unified, consolidated, and combined approach in the provision of medical care to distinguish it from integrative medicine, in which one provides allopathic, complementary, or holistic therapies.

When we consider integrated medicine, clinical experience enables scientific evidence, and scientific evidence empowers clinical experience. After all, we are better positioned to make seminal observations in the clinic rather than in the laboratory. This should make us more inclined to frame pertinent scientific hypotheses and adhere to the scientific method, i.e., to conduct translational research in the proper sequence—from the bedside to the bench rather than from the bench to the bedside.

### 6.1. Clinical Risk 

Let us examine the role of integrated vs. genomic medicine in one of the most common and representative solid malignancies, namely breast cancer. In early-stage hormone-receptor-positive breast cancer, genomic assays are in wide clinical use. When we are preoccupied with the idea that breast cancer patients with high genomic risk (and high clinical risk) on a 70 gene assay would benefit from chemotherapy, we tend to be oblivious to the fact that the same data also shows that those with high genomic risk but low clinical risk do not benefit from chemotherapy [43,44]. 

### 6.2. Recurrence Score 

Perhaps clinical risk trumps genomic risk. Hence, targeted anti-HER2 therapy for HER2+ breast cancer is inadequate treatment—it must still be combined with chemotherapy, despite HER2 amplification being identified as an oncogenic driver [45,46,47]. When it concerns triple negative breast cancer for which chemotherapy is an integral treatment, we do not even bother to check for recurrence score in genomic assays [48,49]. Unfortunately, it is habitual to ignore data that do not match with our standard hypothesis, especially when we do not have a pertinent hypothesis to account for the observations. 

### 6.3. Risk Stratification 

When treatment is curative, clinical risk (clinical stage and tumor biomarker level) may override genomic risk (presence of isochromosome 12p, i(12p)) for the purposes of risk stratification in germ cell tumor of the testis (TGCT). Hence, we cure embryonal carcinoma (with chemotherapy) and teratoma (with surgery), both of which are i(12p)+ in a mixed TGCT. Although i(12p) has diagnostic value, it does not provide any prognostic or predictive utility for risk stratification in TGCT [50,51]. Similarly, when treatment is effective, genomic risk (deletion or loss of chromosome 5 or 7) becomes less pertinent, as is the case with venetoclax + azacitabine or decitabine treatment for acute myelogenous leukemia (AML) [52]. 

### 6.4. Minimal Residual Disease 

In the case of acute lymphocytic leukemia, minimal residual disease (MRD) trumps all other prognostic factors [53]. However, is MRD more accurately defined by the cancer genome or by its stem-ness origins and stem-like properties? This central question affects how we design drug versus therapy development. It has major implications regarding timing and mixing of treatments, and how we implement consolidation and maintenance therapies in cancer care.

### 6.5. Multimodal Therapy 

In many respects, we are already practicing integrated medicine. We have cured many patients with TGCT by means of multimodal therapy, e.g., chemotherapy and surgery/radiation therapy, without resorting to their tell-tale genetic signature, namely i(12p) [54,55]. After all, a germ cell is a primordial stem cell. TGCT is a prototype stem-ness cancer. It takes a multimodal (or integrated) approach to cure a heterogeneous tumor: controlling the chemo-sensitive systemic components (e.g., embryonal carcinoma with chemotherapy) and the chemo-resistant localized compartments (e.g., teratoma with surgery), managing both the metastatic and dormant constituents, and treating the cancer itself as well as its ubiquitous microenvironment. 

It is hard to imagine that genomic medicine and targeted therapy will accomplish the monumental feat of curing a solid cancer. After all, we can only control (not cure) a solid cancer with genomic medicine before it becomes resistant again and develops an escape mechanism to the treatment. We will always need to regroup and discover yet another genetic target and design another targeted therapy to control an intractable tumor and delay the inevitable. 

Importantly, when we accept an alternative stem cell theory about the origin and nature of cancer, we will need to adopt a different practice and adapt to a different culture of cancer care. According to a stem cell theory of cancer, we may need to replace genomic (or precision) medicine with integrated medicine and substitute targeted therapy with multimodal therapy to elevate cancer research and increase cancer cures.

## 7. Therapy Development

Even though we promote drug development, we should not ignore therapy development [50,51]. Again, the distinction between therapy development and drug development reveals a major difference between heuristic, integrated approaches and reductionist, isolated approaches, which affects our conduct of sound scientific research and discovery of beneficial clinical treatments.

Perhaps we have glorified drug development at the expense of therapy development. Although an arsenal of drugs is indispensable for our management of cancer, knowing how and when to use those drugs in the right patients and for the right cancer types is the mantra of therapy development. 

Importantly, navigating therapy versus drug development affects how we deliver integrated versus precision medicine and dispense multimodal versus targeted therapy to maximize clinical outcomes and optimize patient care. 

We need to be cognizant and vigilant that the reductionist method is prone to generation of one-way, bench-to-bedside translational research that produces precision medicine in contrast to integrated medicine. Without question, the labor from these efforts has borne fruits, including targeted therapy and immunotherapy for the treatment of innumerable cancers. Without doubt, the products of translational research, precision medicine, and targeted therapy have attenuated the clinical course of certain diseases and ameliorated the quality and longevity of life for many patients.

Nevertheless, translational research, precision medicine, and targeted therapy have their share of limitations, shortcomings, and disappointments. Recurrence of cancer is common. Resistance to treatment seems inevitable. No one denies that precision medicine and targeted therapy are less likely, if not unlikely, to be curative. Yet, the oncology community is reticent when we need to combine precision medicine or targeted therapy with imprecise and indiscriminate chemotherapy to improve clinical outcomes. 

As translational research may be at the root of a preponderance of our failures in drug development and data reproducibility, perhaps it is time to reconsider its value in cancer research and cancer care. We propose that one way to enhance the traditional translational research is to make sure that it is a two-way process. Importantly, the first step should be from bedside to bench, not bench to bedside. It is a giant step that is likely to be transformative when it concerns therapy versus drug development as well as data reproducibility versus irreproducibility. 

When we eulogize or criticize translational research, we intend to prioritize therapy over drug development, integrated medicine over precision medicine, and multimodal therapy over targeted therapy in patient care. We recognize that cancer is more complex than precise. We realize that we need to treat the whole tumor in a real patient in the clinic rather than parts of the tumor in an experimental model in the laboratory.

## 8. Limitations and Drawbacks

Unlike finding genetic mutations, identifying stem cells can be elusive. There will be technical and technological challenges in the study of stem cells when the very nature of these multifaceted cells (e.g., being dynamic and dormant) is also dependent on a similarly fluid and intricate niche. One way to overcome this limitation is to devise unique panels of molecular signatures aligned with distinct niches rather than specific biomarkers alone to elucidate a hierarchical order of various stem cell lineages.

Exceptional clinical observations are a rarity. If we rely on them in our heuristic approach and if we need them to lay the foundation of the scientific method and translational research, then we need some good fortune to recognize one when we face it. One way to overcome this limitation is to be primed and prepared for a paradigm-shifting clinical observation when the opportunity arises. Otherwise, it is more than likely that we will miss an exceptional clinical observation, because without a proper theory we may not be cognizant of it, and we will overlook it even when it is right in front of our eyes. 

A potential drawback with a stem cell theory of cancer is that targeting stem-ness in a cancer can be hazardous and risky if stem-ness is also inherent and prevalent in normal tissues and cells. Would inadvertent stem cell therapy that replaces the right stem cells in the wrong places instigate malignancy? Would indiscrete therapy that tackles stem-ness pathways and factors incite autoimmunity? A proper theory ensures that we accept and learn from the reality of cancer. For example, if we cannot cure certain cancers or eradicate cancer stem cells, we mitigate it—by attenuating their microenvironment and keeping them differentiated, dormant, and innocuous. 

Another inevitable drawback with a stem cell theory of cancer relates to its prediction about the very origin and nature of cancer itself—tumor heterogeneity. Unlike a genetic theory of cancer, a stem cell theory of cancer anticipates and explicates tumor heterogeneity, which makes a mixed tumor with multiple pathological phenotypes, myriad cellular components, and the ubiquitous microenvironment a dilemma to study and a challenge to treat. Ironically, one way to overcome this shortcoming and an offshoot of the stem cell (vs. genetic) theory of cancer is to develop integrated (vs. precision) medicine, and to design multimodal (vs. targeted) therapy. In the process, we also reduce failures in drug development and data reproducibility.

## 9. Conclusions

In many respects, our current scientific culture and conventional reductionist approach is the brainchild of translational research, genomic medicine, and targeted therapy. It is also the breeding ground for our miscues in drug development and misgivings in data reproducibility. 

Importantly, a heuristic approach, integrated medicine, and multimodal therapy may be our blueprint to keep scientific research vibrant and the scientific method honest, to resolve rather than cause more snags in drug development and data reproducibility, and to enhance therapy development and patient care. 

**Figure 1:**
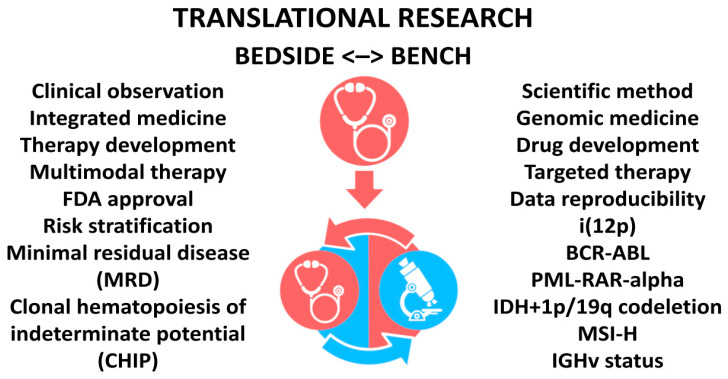
Stem cell theory of cancer and translational research: Biological processes and clinical implications. Illustration by Benjamin Tu.

A stem cell theory of cancer embraces genomic medicine and empowers integrated medicine. It may elevate cancer research by emending translational research and enforcing the scientific method. It may improve cancer care by enabling targeted therapy and employing a multimodal strategy (Figure 1). We will heal the whole patient and treat the whole cancer.

Einstein was right when he said that we need to have the right theory to make the right observation. If he were an oncologist, he would probably also say that we need to make the right observation in the clinic and then test the right theory in both the clinic and the laboratory (Figure 1).

## Data Availability

The data presented are available in the references cited in this article.
